# Supplementary material for: Atomic structures of respiratory complex III2, complex IV, and supercomplex III2-IV from vascular plants
Source: eLife. 2021 Jan 19;10:e62047. doi: 10.7554/eLife.62047 (PMC7815315; doi:10.7554/eLife.62047)
Supplement: Supplementary file 1. [file elife-62047-supp1.docx]

**Supplementary File 1**

Atomic structures of respiratory complex III_2_, complex IV and supercomplex III_2_-IV from vascular plants

**Supplementary File 1a.** Mass spectrometry identification of *V. radiata* CIII and CIV subunits.

**Supplementary File 1b.** Model building statistics by subunit.

**Supplementary File 1c.** CIII_2_ and CIV subunit homologues in plants, yeast and mammals.

**Supplementary File 1d.** RNA edits identified in *V. radiata* CIII_2_ and CIV subunits.

**Supplementary File 1a. Mass spectrometry identification of *V. radiata* CIII_2_ and CIV subunits.**

| ***V. radiata* protein name** | ***V. radiata* gene name** | **Sequence database accession^1^** | **Mass (kDa)** | **# unique peptide fragments** | **% coverage** |
| --- | --- | --- | --- | --- | --- |
| **Complex III** | | | | | |
| MPP-β | LOC106759431 | A0A1S3TWG4 | 59 | 5 | 80% |
| MPP-α | LOC106774328 | A0A1S3VF71 | 54 | 44 | 74% |
| COB | cob | E9KZM1 | 44 | 2 | 6% |
| CYC1 | LOC106765385 | A0A1S3UHP8 | 34 | 11 | 64% |
| UCR1 | LOC106753663 | A0A1S3TB49 | 30 | 30 | 62% |
| QCR7 | LOC106763052 | A0A1S3U9J1 | 14 | 44 | 76% |
| QCR8 | LOC106763113 | A0A1S3U9S5 | 8 | 5 | 49% |
| QCR6 | LOC106775083 | A0A1S3VHC0 | 8 | 15 | 90% |
| QCR9 | LOC106757742 | A0A1S3TQD2 | 8 | 9 | 68% |
| QCR10 | Vradi05g05180 | UPI000FCF06EC | 9 | n.i. | n.i. |
| **Complex IV** | | | | | |
| COX1 | cox1 | E9KZL2 | 57 | 9 | 14% |
| COX2 | LOC106760164 | A0A1S3TZB2 | 43 | 8 | 25% |
| COX3 | cox3 | E9KZN7 | 30 | 5 | 7% |
| COX4 | LOC106757297 | A0A1S3TNX9 | 14 | 10 | 48% |
| COX5b | LOC106760900 | A0A1S3U1E4 | 17 | 12 | 57% |
| COX6a | LOC106766916 | A0A1S3UMA0 | 11 | 10 | 66% |
| COX6b | LOC106778794 | A0A1S3VV60 | 20 | 31 | 60% |
| COX5c | LOC106771138 | A0A1S3V319 | 7 | 3 | 41% |
| COX7a | LOC106759053 | LOC106759053 | 8 | 3 | 73% |
| COX7c | LOC106772338 | LOC106772338 | 10 | 3 | 17% |
| Putative CIV plant-specific subunit previously identified by mass spec^[1]^, not in this structure | | | | | |
| COX-X1 | LOC106769953 | A0A1S3UZ79 | 9 | 2 | 32% |

^1^UniProt database for all sequences except for QCR10, which corresponds to UniParc database.

n.i., not identified

Raw mass spectrometry files and search results are available from Massive (<https://massive.ucsd.edu/>) under code #MSV000086237 and ProteomeExchange (<http://www.proteomexchange.org/>) under code #PXD021850.

**Supplementary File 1b. Model building statistics by subunit.**

| *V. radiata* subunit | Uniprot ID | Chain ID | Total residues | Pre-seq. end^1^ | Atomic residues | % Atomic^2^ | TMH | RNA editing sites^3^ | Ligands, lipids |
| --- | --- | --- | --- | --- | --- | --- | --- | --- | --- |
| **CIII_2_** | | | | | | | | | |
| MPP-b | A0A1S3TWG4 | A | 527 | 40 | 41-527 | 100% | - | - | Zn, 2 lipids |
| MPP-a | A0A1S3VF71 | B | 506 | 49 | 50-506 | 100% | - | - | - |
| COB | E9KZM1 | C | 393 | n.a. | 2-389 | 98% | 8 | Yes | 2 x heme *b*, 9 lipids |
| CYC1 | A0A1S3UHP8 | D | 307 | 62 | 62-307 | 100% | 1 | - | heme *c*_1_ |
| UCR1 | A0A1S3TB49 | E | 271 | 62 | 72-148 | 36% | 1 | - | See note 4 |
| QCR7 | A0A1S3U9J1 | F | 122 | n.a. | 7-122 | 94% | - | - | 1 lipid |
| QCR8 | A0A1S3U9S5 | G | 72 | n.a. | 3.0-72 | 96% | 1 | - | 4 lipids |
| QCR6 | A0A1S3VHC0 | H | 69 | n.a. | 5-69 | 93% | - | - | - |
| QCR9 | A0A1S3TQD2 | J | 72 | n.a. | 10-69 | 82% | 1 | - | 1 lipid |
| QCR10 | UPI000FCF06EC* | K | 81 | n.a. | 11-41 | 37% | 1 | - | - |
| MPP-b | A0A1S3TWG4 | M | 527 | 40 | 41-527 | 100% | - | - | Zn, 3 lipids |
| MPP-a | A0A1S3VF71 | N | 506 | 49 | 49-506 | 100% | - | - | - |
| COB | E9KZM1 | O | 393 | n.a. | 2-389 | 98% | 8 | Yes | 2 x heme *b*, 5 lipids |
| CYC1 | A0A1S3UHP8 | P | 307 | 62 | 62-307 | 100% | 1 | - | heme *c*_1_, 2 lipids |
| UCR1 | A0A1S3TB49 | Q | 271 | 62 | 75-148 | 35% | 1 | - | See note 4; 1 lipid |
| QCR7 | A0A1S3U9J1 | R | 122 | n.a. | 8-122 | 93% | - | - | 1 lipid |
| QCR8 | A0A1S3U9S5 | S | 72 | n.a. | 3.0-72 | 96% | 1 | - | 1 lipid |
| QCR6 | A0A1S3VHC0 | T | 69 | n.a. | 6-69 | 91% | - | - | - |
| QCR9 | A0A1S3TQD2 | V | 72 | n.a. | 11-69 | 81% | 1 | - | - |
| QCR10 | UPI000FCF06EC* | W | 81 | n.a. | 10-41 | 38% | 1 | - | - |
| Total | | | 4840 | n.a. | n.a. | 90% | 13 | 2 subunits | 8 ligands, 30 lipids |
| **CIV** | | | | | | | | | |
| Cox1 | E9KZL2 | a | 527 | n.a. | 1-524 | 99% | 12 | Yes | heme *a*, heme *a*_3_, Cu_B_, Mg, 8 lipids |
| Cox2 | A0A1S3TZB2** | b | 250 | n.a. | 12-250 | 95% | 2 | - | Cu_A_, 4 lipids |
| Cox3 | E9KZN7 | c | 265 | n.a. | 1-265 | 100% | 7 | Yes | 6 lipids |
| Cox4 | A0A1S3TNX9*** | d | 79 | n.a. | 1-76 | 96% | 1 | - | - |
| Cox5b | A0A1S3U1E4 | e | 150 | 34 | 52-145 | 81% | - | - | Zn |
| Cox6a | A0A1S3UMA0 | f | 100 | 29 | 37-95 | 83% | 1 | - | - |
| Cox6b | A0A1S3VV60 | g | 181 | n.a. | 107-180 | 41% | - | - | - |
| Cox5c1 | A0A1S3V319 | h | 64 | n.a. | 14-62 | 77% | 1 | - | - |
| Cox7a | LOC106759053 | i | 67 | n.a. | 2-65 | 94% | 1 | - | 1 lipid |
| Cox7c | LOC106772338 | j | 96 | 29 | 47-93 | 70% | 1 | - | 1 lipid |
| Total | | | 1,779 | n.a. | n.a. | 88% | 26 | 2 subunits | 6 ligands, 20 lipids |

^1^Likely end of the mitochondrial import pre-sequence, based on homology to the corresponding *A. thaliana* subunit wherever possible or the *V. radiata* structure. n.a., not available. Note that COB and COX1-3 are mitochondrially encoded, so they do not contain import sequences.

^2^With respect to the number of residues of the mature protein, i.e. after cleavage of the pre-sequence. If the pre-sequence is not available, the full-length protein was used in the denominator.

^3^Refer to Table 5 for further details. Changes were only made when the cryoEM density was unambiguous.

^4^QCR1 head domain contains an iron-sulfur cluster. However, given that the head domain of UCR1 is not included in the model, the FeS cluster is not present as a ligand.

*UniParc ID.

**The *V. radiata* COX2 Uniprot entry (unreviewed, automatic entry) is mis-annotated: it contains an additional 132 N-terminal residues that are not part of the coding sequence as per the *V. radiata* genome annotation (Legume Information System, https://legumeinfo.org/gb2/gbrowse/Vr1.0). The values reported here correspond to the sequence beginning at Met133 (Uniprot numbering).

***The *V. radiata* COX4 Uniprot entry (unreviewed, automatic entry) is mis-annotated: it contains an additional 45 N-terminal residues that are not part of the coding sequence as per the *V. radiata* genome annotation (Legume Information System, https://legumeinfo.org/gb2/gbrowse/Vr1.0). The values reported here correspond to the sequence beginning at Met46 (Uniprot numbering).

**Supplementary File 1c. CIII_2_ and CIV subunit homologues in plants, yeast and mammals.**

*V. radiata* homologues were obtained by performing BLASTp searches of the *Arabidopsis thaliana* genes^[2]^. Mammalian and yeast homologues were obtained from ref. [3],[4]. Additional BLASTp searches were performed as needed.

| **ChainID^1^** | **Subunit name^2^** | | | | | |  | **Gene^2^** | | | | |
| --- | --- | --- | --- | --- | --- | --- | --- | --- | --- | --- | --- | --- |
|  | ***V. radiata*** | | ***A. thaliana*** | ***S. cerevisiae*** | ***B. taurus*** | |  | ***V. radiata*^3^** | | ***A. thaliana*** | ***S. cerevisiae*** | ***B. taurus*** |
| **Complex III** | | | | | | | | | | | | |
| A,M | MPP-β | | MPP-β | Cor1 | UQCRC1 | |  | LOC106759431 | | At3g02090 | *COR1* | *UQCRC1* |
| B,N | MPP-α | | MPP-α | Cor2 | UQCRC2 | |  | LOC106774328 | | At1g51980 | *QCR2* | *UQCRC2* |
| C,O | COB | | COB | Cytb | MT-CYB | |  | cob | | AtMg00220 | *COB* | *MT-CYB* |
| D,P | CYC1 | | CYC1 | Cytc1 | CYC1 | |  | LOC106765385 | | At5g40810 | *CYT1* | *CYC1* |
| E,Q | UCR1 | | UCR1 | Rip1 | UQCRFS1 | |  | LOC106753663 | | At5g13430 | *RIP1* | *UQCRFS1* |
| F,R | QCR7 | | QCR7 | Qcr7 | UQCRB | |  | LOC106763052 | | At4g32470 | *QCR7* | *UQCRB* |
| G,S | QCR8 | | UCRQ | Qcr8 | UQCRQ | |  | LOC106763113 | | At3g10860 | *QCR8* | *UQCRQ* |
| H,T | QCR6 | | QCR6 | Qcr6 | UQCRH | |  | LOC106775083 | | At1g15120 | *QCR6* | *UQCRH* |
| J,V | QCR9 | | QC9 | Qcr9 | UQCR10 | |  | LOC106757742 | | At3g52730 | *QCR9* | *UQCR10* |
| K,W | QCR10 | | UCRY | Qcr10 | UQCR11 | |  | Vradi05g05180 | | At2g40765 | *QCR10* | *UQCR11* |
| **Complex IV** | | | | | | | | | | | | |
| a | COX1 | | COX1 | Cox1 | COX1 | |  | cox1 | | AtMg01360 | *COX1* | *COX1* |
| b | COX2 | | COX2 | Cox2 | COX2 | |  | LOC106760164 | | AtMg00160 | *COX2* | *COX2* |
| c | COX3 | | COX3 | Cox3 | COX3 | |  | cox3 | | AtMg00730 | *COX3* | *MT-CO3* |
| d | COX4 | | COX-X2 | Cox5a | COX4 | |  | LOC106757297 | | At4g00860 | *COX5a* | *CO4IL1* |
| e | COX5b | | COX5b | Cox4 | COX5B | |  | LOC106760900 | | At3g15640 | *COX4* | *COX5B* |
| f | COX6a | | COX6a | Cox13 | COX6A | |  | LOC106766916 | | At4g37830 | *COX13* | *COX6A1* |
| g | COX6b | | COX6b | Cox12 | COX6B | |  | LOC106778794 | | At1g22450 | *COX12* | *COX6B1* |
| h | COX5c | | COX5c | Cox9 | COX6C | |  | LOC106771138 | | At2g47380 | *COX9* | *COX6C1* |
| i | COX7a | | COX-X4 | Cox7 | COX7A | |  | LOC106759053 | | At4g21105 | *COX7* | *CO7A1* |
| j | COX7c | | COX-X3 | Cox8 | COX7C | |  | LOC106772338 | | At1g72020 | *COX8* | *COX7C* |
| - | - | | - | Cox6 | COX5A | |  | - | | - | *COX6* | *COX5A* |
| - | - | | - | Cox26 | - | |  | - | | - | *COX26* | - |
| - | - | | - | - | COX7B | |  | - | | - | - | *COX7B* |
| - | - | | - | - | COX8B | |  | - | | - | - | *COX8B* |
| Putative CIV plant-specific subunits identified in previous mass spec studies[1, 5] not seen in our structure | | | | | | | | | | | | |
| *A. thaliana* gene | | *A. thaliana*  protein | | | | *V. radiata* gene | | | *V. radiata* protein | | | |
| At5g27760 | | COX-X1 | | | | LOC106769953 | | | uncharacterized protein LOC106769953^4^ | | | |
| At3g43410 | | COX-X5 | | | | LOC106769343 | | | ARF guanine-nucleotide exchange factor GNL2 | | | |
| At3g12150 | | a/b hydrolase family protein | | | | LOC106756702 | | | protein ABHD18 | | | |
| At4g14570 | | Acyl-amino-acid-releasing enzyme | | | | LOC106772197 | | | acylamino-acid-releasing enzyme | | | |
| At4g00960 | | Putative receptor-like protein kinase | | | | LOC106771829 | | | putative receptor-like protein kinase | | | |

^1^ *V. radiata* chain ID in our model.

^2^ For subunits with multiple isoforms, only one is noted.

^3^ Accessed on Uniprot, except for QCR10, which was accessed from EnsenblGenome with its UniParc ID.

^4^ Identified in our mass spectrometry.

**Supplementary File 1d. RNA edits identified in *V. radiata* CIII_2_ and CIV subunits.** RNA editing sites were initially identified by an unambiguous mismatch in the cryoEM density and the expected density for the mitochondrial-DNA-encoded residue. The existence of the RNA editing site in other plants, or the implied restoration of the consensus sequence was then inspected. Amino acid changes to the atomic model (with respect to the mt-DNA sequence) were only made for amino acid positions that had unambiguous cryoEM density evidence and whose editing is conserved or would restore the conserved sequence.

| **Subunit** | ***V. radiata*** | | | **Other species(*)** |
| --- | --- | --- | --- | --- |
|  | **Position** | **mt-DNA residue** | **Edited in model to:** | **Identical edit or consensus restoration:** |
| COB | 96 | L | F | At, potato, wheat, rice^[6-9]^ |
|  | 100 | H | Y | Potato, wheat, rice^[6, 7]^ |
|  | 109 | H | Y | At, potato, wheat, rice^[6-9]^ |
|  | 120 | R | W | Potato, wheat, *Chlamydomonas reinhardtii*^[6]^ |
|  | 190 | H | Y | At, potato, wheat, rice^[6-9]^ |
|  | 270 | P | S | Potato, wheat, rice^[6, 7]^ |
|  | 285 | H | Y | At, potato, wheat, rice^[6-9]^ |
|  | 303 | P | L | At, potato, wheat, rice^[6-9]^ |
|  | 328 | H | Y | At, potato, wheat, rice^[6-9]^ |
|  | 339 | R | C | At^[8, 9]^ |
|  | 361 | P | S | Potato, rice^[6, 7]^ |
|  | 375 | P | L | Potato, wheat, rice^[6, 7]^ |
| COX1 (***) | 81 | S | F | (**) At, Pd, Sc, Hs, Bt |
|  | 85 | S | F | (**) At, Pd |
|  | 148 | S | L | (**) At, Pd, Sc, Hs, Bt |
|  | 151 | S | F | (**) At, Pd, Sc, Hs, Bt |
|  | 172 | S | F | (**) At |
|  | 192 | P | L | (**) At, Hs |
|  | 197 | P | L | (**) At, Sc, Hs, Bt |
|  | 223 | S | F | Variety of vascular and non-vascular plants^[10]^ |
|  | 239 | R | W | Variety of vascular and non-vascular plants^[10]^ |
|  | 249 | P | L | Variety of vascular and non-vascular plants^[10]^ |
|  | 254 | S | F | Variety of vascular and non-vascular plants^[10]^ |
|  | 396 | H | Y | (**) At, Pd, Sc |
|  | 427 | L | F | Variety of vascular and non-vascular plants^[10]^ |
|  | 469 | R | C | (**) At |
| COX3 | 82 | P | L | At, wheat, magnolia, olive^[8, 9, 11-13]^ |
|  | 86 | S | F | At, wheat, magnolia, olive^[8, 9, 11-13]^ |
|  | 88 | P | L | Magnolia^[13]^ |
|  | 102 | R | W | Magnolia, olive^[12, 13]^ |
|  | 104 | S | F | At, wheat, magnolia, olive^[8, 9, 11-13]^ |
|  | 105 | S | F | At, wheat, magnolia, olive^[8, 9, 11-13]^ |
|  | 138 | P | L | At, wheat, magnolia, olive^[8, 9, 11-13]^ |
|  | 171 | S | L | Wheat, magnolia^[11, 13]^ |
|  | 252 | R | W | At, wheat, magnolia, olive^[8, 9, 11-13]^ |

(*) Non-exhaustive list.

(**) RNA edit restores identity to the mt-DNA-encoded residue to the indicated model organisms (non-exhaustive): *Arabidopsis thaliana* (At), *Paracoccus denitrificans* (Pd), *Saccharomyces cerevisiae* (Sc), *Bos taurus* (Bt), *Homo sapiens* (Hs).

(***) *A. thaliana* does not have any RNA editing sites in cox1; all the RNA editing sites identified in the other species are already mt-DNA mutations in *A. thaliana*.

**Supplementary References**

1. Millar, A.H., et al., *Mitochondrial cytochrome c oxidase and succinate dehydrogenase complexes contain plant specific subunits.* Plant Mol Biol, 2004. **56**(1): p. 77-90.

2. Meyer, E.H., E. Welchen, and C. Carrie, *Assembly of the Complexes of the Oxidative Phosphorylation System in Land Plant Mitochondria.* Annu Rev Plant Biol, 2019. **70**: p. 23-50.

3. Hartley, A.M., et al., *Structure of yeast cytochrome c oxidase in a supercomplex with cytochrome bc1.* Nat Struct Mol Biol, 2019. **26**(1): p. 78-83.

4. Marechal, A., et al., *Yeast cytochrome c oxidase: a model system to study mitochondrial forms of the haem-copper oxidase superfamily.* Biochim Biophys Acta, 2012. **1817**(4): p. 620-8.

5. Senkler, J., et al., *The mitochondrial complexome of Arabidopsis thaliana.* Plant Journal, 2017. **89**(6): p. 1079-1092.

6. Zanlungo, S., et al., *RNA editing of apocytochrome b (cob) transcripts in mitochondria from two genera of plants.* Curr Genet, 1993. **24**(4): p. 344-8.

7. Ito, Y., et al., *RNA editing of transcripts of the gene for apocytochrome b (cob) in rice mitochondria.* Genes Genet Syst, 1996. **71**(2): p. 85-9.

8. Giege, P. and A. Brennicke, *RNA editing in Arabidopsis mitochondria effects 441 C to U changes in ORFs.* Proc Natl Acad Sci U S A, 1999. **96**(26): p. 15324-9.

9. Bentolila, S., L.E. Elliott, and M.R. Hanson, *Genetic architecture of mitochondrial editing in Arabidopsis thaliana.* Genetics, 2008. **178**(3): p. 1693-708.

10. Sper-Whitis, G.L., J.L. Moody, and J.C. Vaughn, *Universality of mitochondrial RNA editing in cytochrome-c oxidase subunit I (coxI) among the land plants.* Biochim Biophys Acta, 1996. **1307**(3): p. 301-8.

11. Gualberto, J.M., J.H. Weil, and J.M. Grienenberger, *Editing of the wheat coxIII transcript: evidence for twelve C to U and one U to C conversions and for sequence similarities around editing sites.* Nucleic Acids Res, 1990. **18**(13): p. 3771-6.

12. Perrotta, G., A. Cavallotti, and C. Quagliariello, *Reduced requirement for RNA editing in the mitochondrial cox3 transcript of Olea europaea L.* Curr Genet, 1997. **31**(2): p. 185-9.

13. Perrotta, G., et al., *RNA editing in the cox3 mRNA of Magnolia is more extensive than in other dicot or monocot plants.* Biochim Biophys Acta, 1996. **1307**(3): p. 254-8.
